# Antimicrobial and Anti-Quorum Sensing Activities of Phlorotannins From Seaweed (*Hizikia fusiforme*)

**DOI:** 10.3389/fcimb.2020.586750

**Published:** 2020-10-30

**Authors:** Jiali Tang, Wenqian Wang, Weihua Chu

**Affiliations:** School of Life Science and Technology, State Key Laboratory of Natural Medicines, China Pharmaceutical University, Nanjing, China

**Keywords:** phlorotannins, anti-quorum sensing, virulence factors, biofilm, *Pseudomonas aeruginosa*, *Caenorhabditis elegans*, anti-infection

## Abstract

Multidrug-resistant bacteria (MDR) are becoming a global health problem, and scientists are continuously investigating new strategies to fight against MDR. Seaweeds are an important source of biological compounds and can serve as natural sources for bacterial infection control. This study evaluated the antimicrobial and anti-quorum sensing (QS) activities of phlorotannins from *Hizikia fusiforme*. The phlorotannins exhibited antimicrobial activity against selected bacterial pathogens and inhibited QS activity of the reporter strain *Chromobacterium violaceum* 12472 by inhibiting purple pigment production. Phlorotannins can decrease the bacterial motility, reduce the production of extracellular protease, hemolysin, and pyocyanin and inhibit biofilm formation of *Pseudomonas aeruginosa*. *In vivo* studies showed that phlorotannins can reduce *P. aeruginosa* inflicted mortality in *Caenorhabditis elegans*. This study shows that phlorotannins from *H. fusiforme* have certain antimicrobial and anti-quorum sensing activities and have the potential to control bacterial infection for pharmaceutical usage.

## Introduction

Antibiotics have been used for bacterial infection treatment for decades, with the majority used as feed additives for animal husbandry (Van Boeckel et al., 2017). However, antibiotics are becoming ineffective because of the emergence of antibiotic-resistant strains of bacteria (Mazer-Amirshahi et al., 2017; Ghosh et al., 2019; Mulani et al., 2019). The emergence and spread of multidrug-resistant bacteria (MDR) have potentially profound consequences for public health in low- and middle-income countries (Van Boeckel et al., 2019). Thus, scientists have made efforts to find new agents and alternative antibiotics to work against bacterial pathogens (Czaplewski et al., 2016; Romero-Calle et al., 2019).

Quorum sensing (QS) is a cell-to-cell communication pathway in microorganisms, in which the expression of several genes, often associated with virulence factors and biofilm formation, is controlled *via* the production and detection of signal molecules in a population density-dependent manner (Rutherford and Bassler, 2012). Furthermore, signal disruption referred to as quorum quenching has been described in several biological systems and is being explored as a novel approach to fight bacterial infections. Disruption of QS is a competition strategy used by microorganisms and higher organisms (Defoirdt, 2018; Fleitas Martínez et al., 2019).

Seaweeds are non-vascular, photosynthetic plants that inhabit near-coastal regions and have been one of the richest and most promising sources of primary and secondary bioactive metabolites with antimicrobial properties (Pérez et al., 2016; Zerrifi et al., 2018). Phlorotannins are polyphenolic compounds of seaweeds and have many biological activities including antioxidant, antidiabetic, anti-inflammatory, and antimicrobial (Javed et al., 2020). In this study, we investigated the effects of phlorotannins from brown seaweed (*Hizikia fusiforme*) on QS activity in *Chromobacterium violaceum* and virulence factors and biofilm formation in *Pseudomonas aeruginosa*. Furthermore, we determined the survival rate of *Caenorhabditis elegans* against *P. aeruginosa* infection.

## Materials and Methods

### Chemicals and Reagents

Phlorotannins were purchased from the Shandong Jiejing Group Corporation (Shandong, China), which were isolated from *Hizikia fusiforme*. The phlorotannins were dissolved in water and stored at 4°C for further experiments. All other chemicals were of reagent grade from commercial sources.

### Biosensors and Culture Conditions

*Chromobacterium violaceum* ATCC 12472, which can produce violacein (purple pigment) under the QS system, was used as a report strain to detect QS inhibition ability (Mclean et al., 2004). The reporter bacterium *Agrobacterium tumefaciens* A136 (pCF218)(pCF372) was used to detect the AHLs of *Pseudomonas aeruginosa*. The antimicrobial activity of phlorotannins was determined against *Staphylococcus aureus* ATCC 25923, *Escherichia coli* ATCC 25922, *Pseudomonas aeruginosa* PAO1, *Aeromonas hydrophila* YJ-1, and *Vibrio parahaemolyticus* X-1.

All bacteria were grown in Luria–Bertani broth (LBB, 0.5% yeast extract, 1.0% tryptone, and 0.5% NaCl, at pH 7.4). *P. aeruginosa*, *S. aureus*, and *E. coli* were grown at 37°C; *C. violaceum*, *A. hydrophila*, and *V. parahaemolyticus* were grown at 30°C. Both the sets of microorganisms were incubated at 120 rpm for 18–24 h. The bacterial density was measured using a spectrophotometer at a wavelength of 600 nm (OD_600_). For long term storage, bacterial cultures were preserved at −70°C in LBB containing 20% (v/v) glycerol.

*Caenorhabditis elegans* N2 (Bristol) was obtained from the Caenorhabditis Genetics Center (CGC). The N2 strain was propagated under standard conditions, synchronized using hypochlorite bleaching, and cultured on nematode growth medium (NGM) at 16–25°C using an *E. coli* strain OP50 as a standard food source.

### AHLs Extraction and Analytical Thin-Layer Chromatography

To evaluate the profiles of AHLs, bacterial culture supernatants were extracted and subjected to analytical thin-layer chromatography (TLC) (Shaw et al., 1997). A 10-ml sample of culture supernatant was extracted twice with equal volumes of ethyl acetate and then dried in a fume hood. The residues of extraction were then dissolved in 100 µl of HPLC-grade ethyl acetate. Analytical TLC was performed on C18 reversed-phase TLC plates (Whatman, Clifton, NJ, USA). Chromatograms were developed with methanol: water (60:40, v:v) then air-dried in a fume hood. TLC plates were overlaid with a culture of the reporter bacterium *A. tumefaciens* A136 (pCF218)(pCF372) seeded in a thin layer of agar containing X-Gal. This traG::lacZ/traR reporter detects 3-oxo-substituted AHL derivatives with acyl chain length from 4 to 12 carbons (*e.g.*, OdDHL). After incubation of the plate at 30°C for 24 h, AHLs were located as green spots on a white background. The development of blue spots indicated the induction of *ß*-galactosidase expression in the reporter strain caused by the presence of AHLs. All the experiments were performed at least twice.

### Determination of Minimum Inhibitory Concentration

The phlorotannins MIC for the selected bacteria was determined using a twofold dilution method in LBB with an inoculum of 10^6^ CFU/ml. After culturing at 37°C for 18 h, the clarity of each tube was observed. The MIC was defined as the lowest concentration at which there is no visible bacteria growth in the tube (Wiegand et al., 2008). The sub-MIC (concentrations below MIC value) of phlorotannins was taken to evaluate the anti-QS activity on *P. aeruginosa*.

The synergistic effect of antibiotics (Levofloxacin and Amikacin) in combination with phlorotannins was tested by the chequerboard titration method as described by He et al. (2014). The final concentrations of the drugs were prepared in a range of 1/16–2 × MIC. The fractional inhibitory concentration index (FICI) was equal to (MIC of antibiotics in combination/MIC of antibiotics alone) + (MIC of phlorotannins in combination/MIC of phlorotannins alone). The definitions of synergism, indifference and antagonism were interpreted as FICI ≤0.5, >0.5 and ≤4.0, and >4.0, respectively.

### Bioassay for QS Inhibition Activity

*C. violaceum* 12472 was used as the biosensor to detect anti-QS activity. Ten milliliters of 50°C warm molten LB soft agar (0.7% agar) was seeded with 20 μl of 18 h cultured *C. violaceum* 12472 and mixed. The mixed culture was poured over the surface of a solidified LB plate to form the overlay. Subsequently, 50 mm wells were punched through the agar, and 100 μl of phlorotannins was loaded into each well. The plates were then cultured at 30°C for 16–18 h. The absence of the purple violacein pigmentation of the bacterial lawn surrounding the well would indicate QS inhibition activity (Chenia, 2013).

### Attenuation of *P. aeruginosa* PAO1 Virulence Factors, Motility, and Biofilm Formation

#### Motility Assay

LB medium with 0.3 and 0.5% (w/v) Difco Bacto-agar was used to characterize the swimming and swarming motility of *P. aeruginosa*. The overnight cultures of *P. aeruginosa* PAO1 were inoculated onto the center of the plates with different sub-MIC phlorotannins using a sterile toothpick. The plates were then incubated at 37°C for 16–18 h, and the motility was assessed by measuring the migration of bacteria from the inoculation point (Yang et al., 2017). The motility was detected in triplicate.

#### Proteolytic and Hemolytic Activities and Pyocyanin Production

Pyocyanin is a blue-redox extracellular product, which is controlled by rhlR-rhlI, lasR-lasI, and PQS QS systems in *P. aeruginosa* (Liang et al., 2008; Wiegand et al., 2008). An overnight culture of *P. aeruginosa* PAO1 cultured with different sub-MIC phlorotannins was centrifuged at 10,000 ×g for 15 min. The supernatant was filtered with a 0.45 μm sterilized film. The supernatant was used for extracellular proteolytic and hemolytic activities and pyocyanin production assay. The proteolytic activity was detected by the method of Nicodème et al. (2005), using 1.0% skim milk as a substrate. The hemolytic activity test was performed using 5% (v/v) sheep blood in a nutrient agar plate (Castro-Escarpulli et al., 2003). Each measurement was conducted in triplicate. Pyocyanin production was measured using the methods of Michel et al. (2002). Two milliliters of cell-free supernatants were extracted with 1.5 ml chloroform and vortexed immediately. After centrifugation (10,000 ×g, 5 min) the top layer was transferred to a new tube where 1.5 ml 0.2 M HCl was added, and the color of the liquid changed to pink. The absorbance was the detected at OD_520_.

#### Biofilm Formation Assay

Quantifying biofilm formation of *P. aeruginosa* was detected as previously described by Agarwala et al. (2014). Using 10 μl of 18 h cultured *P. aeruginosa* PAO1 (10^6^ CFU/ml), diluted with 2 ml LB liquid medium containing a series of sub-MIC phlorotannins, incubation was conducted for 18 h in a static culture. The culture was discarded after incubation, and the tube was washed thrice with Phosphate Buffered Saline (PBS) and fixed in formaldehyde (10%) for 10 min. The solutions were removed, air dried at room temperature, and crystal violet (0.1% in ethanol) was added to stain the biofilm for 15 min. Deionized water was used to wash the unbound dye three times, and the absorbed dye was eluted with ethanol. Then the OD_590_ was recorded, repeated independently three times.

The biofilm formation on coverslips was observed as described by Mishra et al. (2020). Biofilm of *P. aeruginosa* PAO1 was formed on the coverslips (10 × 10 mm) in 12-wells polystyrene microtiter plates (Liu et al., 2019). Different sub-MIC of phlorotannins was added. The inoculated broths were then cultivated for 24 h. The coverslips were washed gently with PBS to remove the un-attached cells. For light microscopic analysis, the coverslips were stained with 0.4% crystal violet (CV) for 10 min. For fluorescence microscope analysis, the coverslips were stained with Live & Dead Bacterial Staining Kit (Shanghai Yeasen Biotech Co. Ltd., Shanghai, China) at room temperature for 15 min in the dark and then washed with PBS to remove excess stain. The coverslips were allowed to dry and visualized under respective microscopes.

#### *C. elegans* Killing Assay

NGM was used to culture the *C. elegans* N_2_, and the protective effect of phlorotannins on *P. aeruginosa* PAO1 infection was evaluated. Treatment plates with different concentrations of phlorotannins were prepared by spreading 100 μl of overnight cultured PAO1. The plate was incubated at 37°C for 16–18 h to grow a bacterial lawn, and *E. coli* OP50 plates were used as a negative control. About 20–30 synchronized L4 worms were transferred to each plate with the bacterial lawn, and the number of worms killed was recorded over 6 days using visual observation of the plates under a microscope (Kirienko et al., 2014).

### Statistical Analysis

All tests were conducted in triplicate and data were presented as the mean values. Analysis of variance was conducted, and differences between the mean values were evaluated for significance using one-way ANOVA with Tukey test correction on the SPSS Statistics 20.0 and Origin Pro 8.0. Differences with a *p* < 0.05 were considered statistically significant.

## Results

### Antibacterial and Anti-QS Activities Assays

The phlorotannins’ antibacterial activity was investigated using a double broth dilution method, and the minimal inhibitory concentration value was calculated. The MIC of phlorotannins against the selected bacteria was: 0.1943 g/ml for *C. violaceum* and *E. coli*; whereas it was 0.0972 g/ml for *S. aureus*, *A. hydrophilia*, *V. parahaemolyticus*, and *P. aeruginosa*.

**Figure 1:**
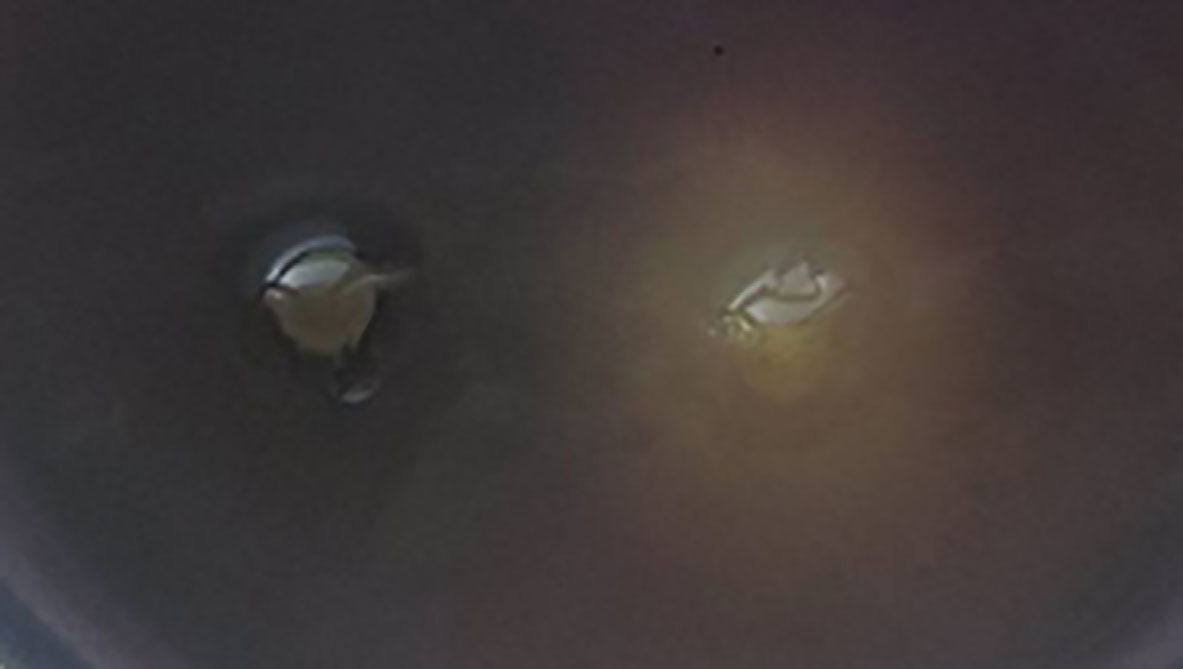
Anti-QS activity of phlorotannins on a biosensor plate containing *C. violaceum* 12472.

Therefore, phlorotannins show QS inhibitory activity in *C. violaceum* 12472. Thus, we used 1/2 MIC to detect the anti-QS effect; after a 16–18 h culture, a muddy zone without purple pigment diameter was observed (**Figure 1**), where the halo zone formation indicates anti-QS phlorotannin activity.

**Figure 2:**
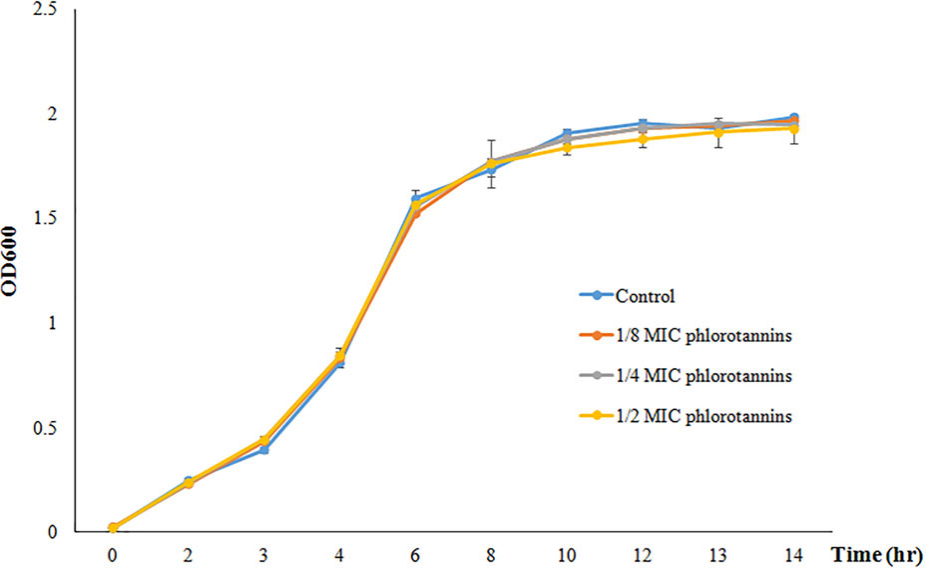
Growth curve of *P. aeruginosa* PAO1 in the presence of different sub-MIC concentrations of phlorotannins (control, 1/8 MIC, 1/4 MIC, and 1/2 MIC).

**Figure 2** showed the growth curve of *P. aeruginosa* PAO1 in the presence of different sub-MICs of phlorotannins; it does not show any significant difference in the growth pattern as compared to phlorotannins’ free group. That indicates that sub-MIC phlorotannins have no effect on the cell division.

**Figure 3:**
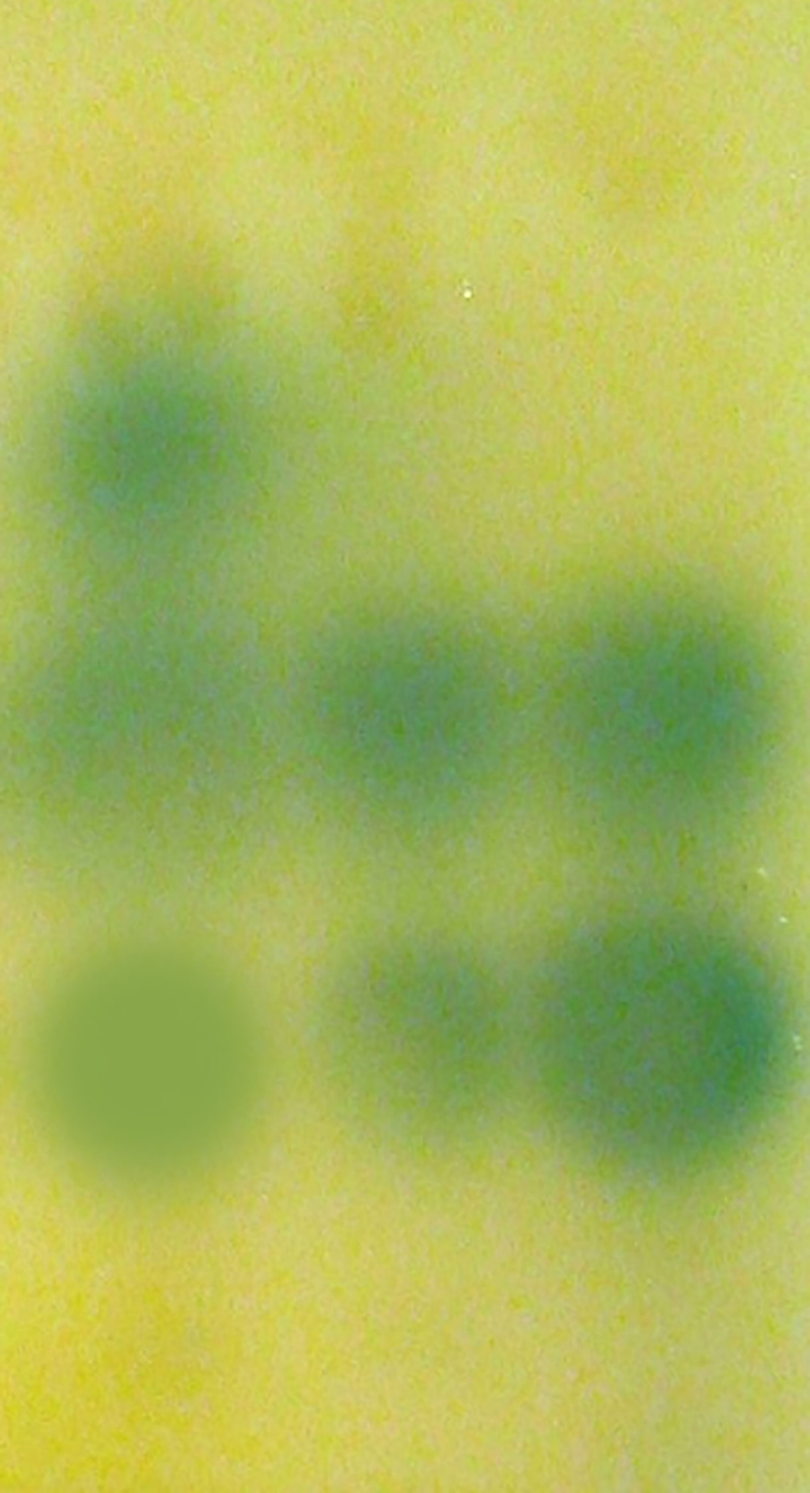
Detection of AHLs by thin layer chromatography (TLC) with the *A. tumefaciens* A136 strain used as a biosensor. Lane 1, Extract of PAO1 culture, lane 2, Extract of PAO1 culture with 1/2 MIC phlorotannins, lane 3, Extract of PAO1 after culture with 1/4 MIC phlorotannins.

Acyl-HSL signal profiles present in *P. aeruginosa* were examined by thin layer chromatography and acyl-HSL-responsive bioassays. We found that phlorotannins did affect acyl-HSL production by *P. aeruginosa*. From **Figure 3**, we can see one of the quorum sensing molecules were lost.

The synergistic effect of antibiotics in combination with phlorotannins was determined. As shown in **Table 1**, the combination of phlorotannins can decreased the MIC of levofloxacin and amikacin. The FICIs were 1.25 and 1.5 for levofloxacin and amikacin, respectively. Thus these results indicated that this combination did not act synergistically against *P. aeruginosa* PAO1.

**Table 1:** Effect of phlorotannins in combination with antibiotics against *P. aeruginosa* PAO1.

Antibiotics	MIC of Antibiotics (mg/L)		MIC of phlorotannins (g/ml)		FICI	Activity
	Alone	Combined with phlorotannins	Alone	Combined with antibiotics		
Levofloxacin	0.25	0.0625	0.0972	0.0972	1.25	indifference
Amikacin	0.5	0.25	0.0972	0.0972	1.5	indifference

### Effect of Phlorotannins on Motility of *P. aeruginosa* PAO1

**Figure 4:**
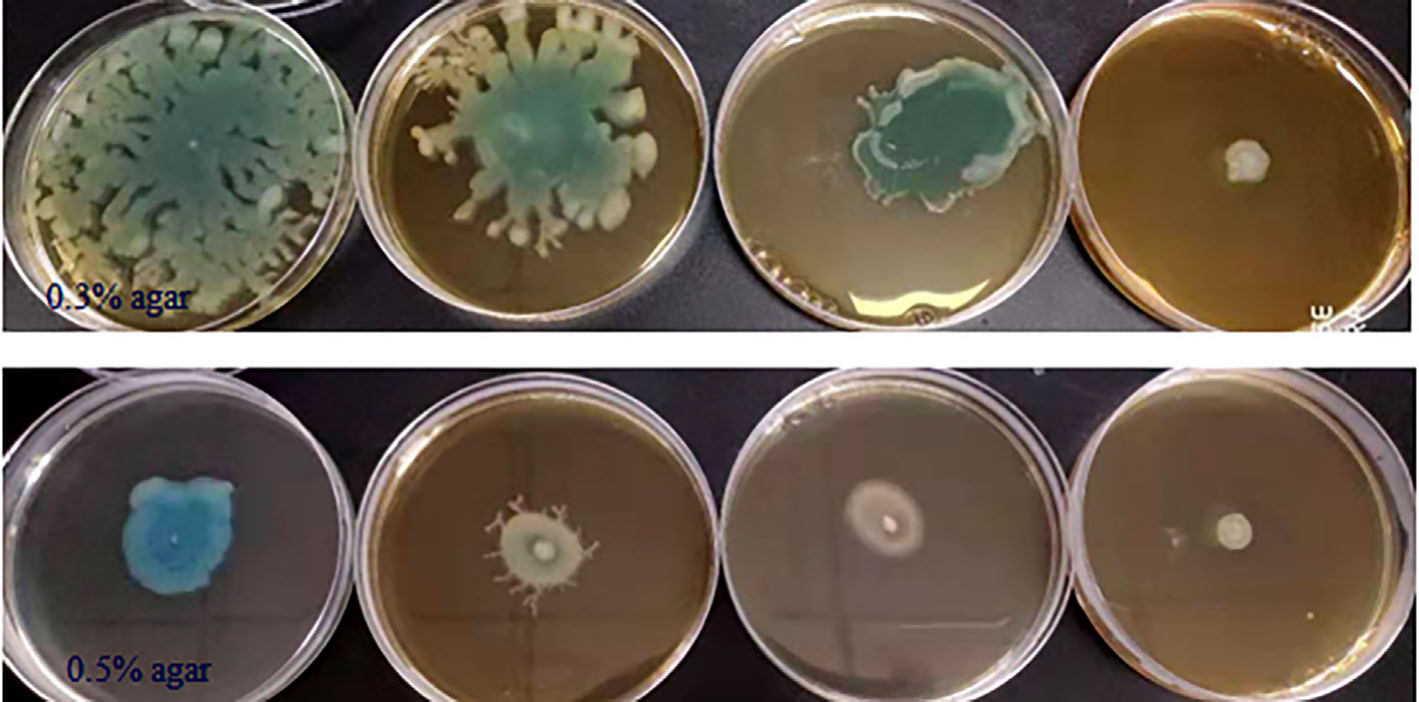
Effect of phlorotannins on the motility of *P. aeruginosa* PAO1. *P. aeruginosa* PAO1 swimming motility and swarming motility on different concentrations of phlorotannins. From left to right: control, 1/8 MIC, 1/4 MIC, and 1/2 MIC.

Some phenotypes in *P. aeruginosa* PAO1, including bacterial motility, are controlled by QS systems; thus we detected the anti-motility activities of phlorotannins on *P. aeruginosa* PAO1. **Figure 4** shows phlorotannins can decrease the motility of *P. aeruginosa* because the diameter of the bacterial lawn decreased with the increasing phlorotannin concentration.

### Phlorotannins Suppress Proteolytic and Hemolytic Activities of *P. aeruginosa* PAO1

The proteolytic and hemolytic activities were detected using skim milk and blood as substrates. The diameters of transparent zones were measured, which indicated the phlorotannins possess proteolytic and hemolytic activities (**Table 2**). Notably, with the increasing phlorotannin concentration, the transparent zone diameter increased. At the highest concentration tested (1/2 MIC, 0.04858 g/ml), the proteolytic activity of *P. aeruginosa* PAO1 reduced by 21.7 and 24.3%, while hemolytic activity reduced by 11.0 and 21.8% when compared to untreated controls.

**Table 2:** Diameters of transparent zone for proteolytic and hemolytic activities.

	Concentration (g/ml)				
Phlorotannins	0	0.00607	0.01214	0.02429	0.04858
Protease	1.52 ± 0.0764	1.26 ± 0.0529	1.21 ± 0.0.513	1.17 ± 0.0289	1.15 ± 0.0500
Hemolysin	1.19 ± 0.0478	1.15 ± 0.0600	1.10 ± 0.0300	1.03 ± 0.0353	0.93 ± 0.0353

Data are expressed as mean ± SE of three separate independent experiments.

### Phlorotannins Decrease Pyocyanin Production of *P. aeruginosa* PAO1

**Figure 5:**
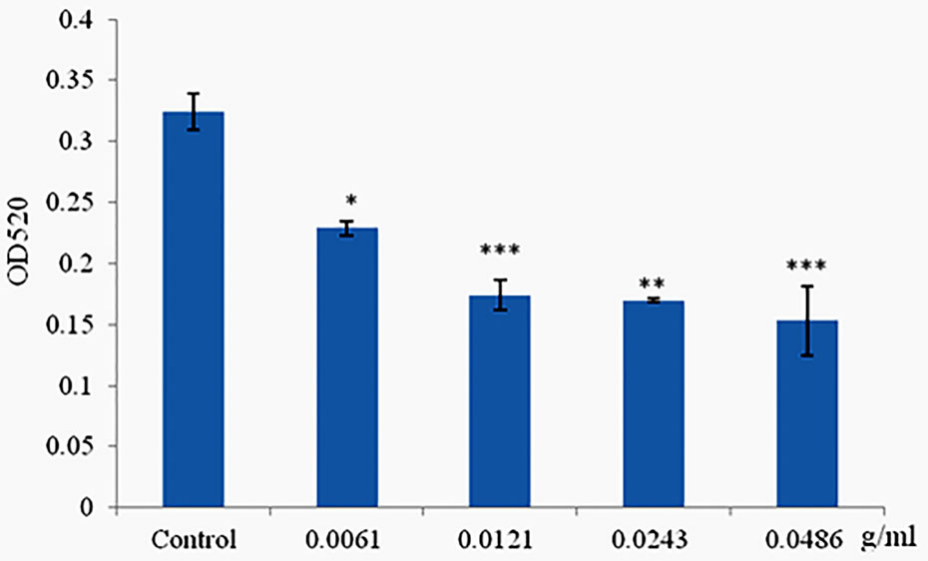
Phlorotannins on pyocyanin production. Asterisks indicate a statistical difference between experimental groups and control groups. Results represent three independent experiments performed in triplicates. (*p < 0.05; **p < 0.01; ***p < 0.001).

Pyocyanin was produced when the cells of *P. aeruginosa* PAO1 reached high density, and production was limited when cultured with materials which can disrupt the QS systems but did not affect cell growth. Phlorotannins significantly reduced pyocyanin production; with the increasing concentration of phlorotannins, the pyocyanin production decreased (**Figure 5**).

### Phlorotannins Inhibit Biofilm Formation of *P. aeruginosa* PAO1

**Figure 6:**
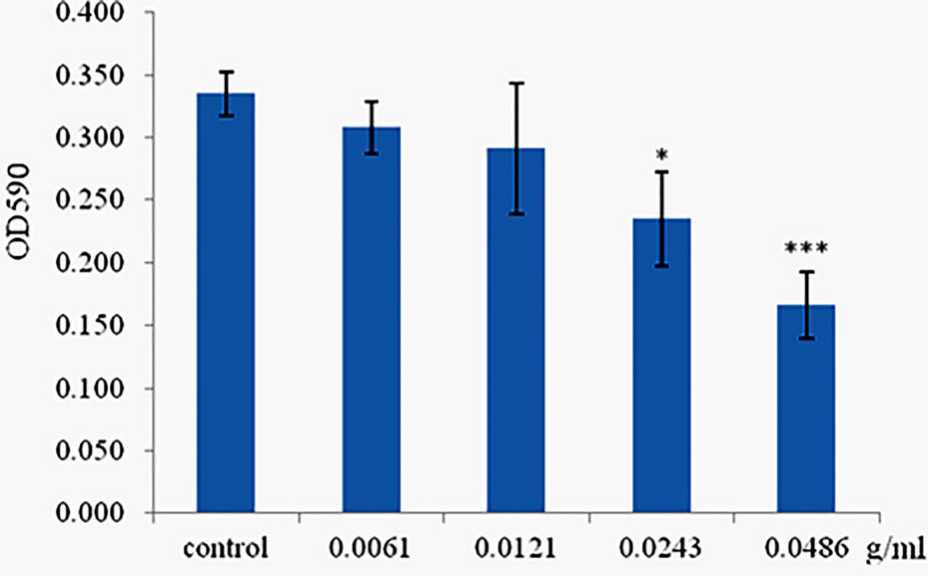
Effect of phlorotannins on biofilm formation. Asterisks indicate a statistical difference between experimental groups and control groups. Results represent three independent experiments performed in triplicates. (*p < 0.05; ***p < 0.001).

**Figure 7:**
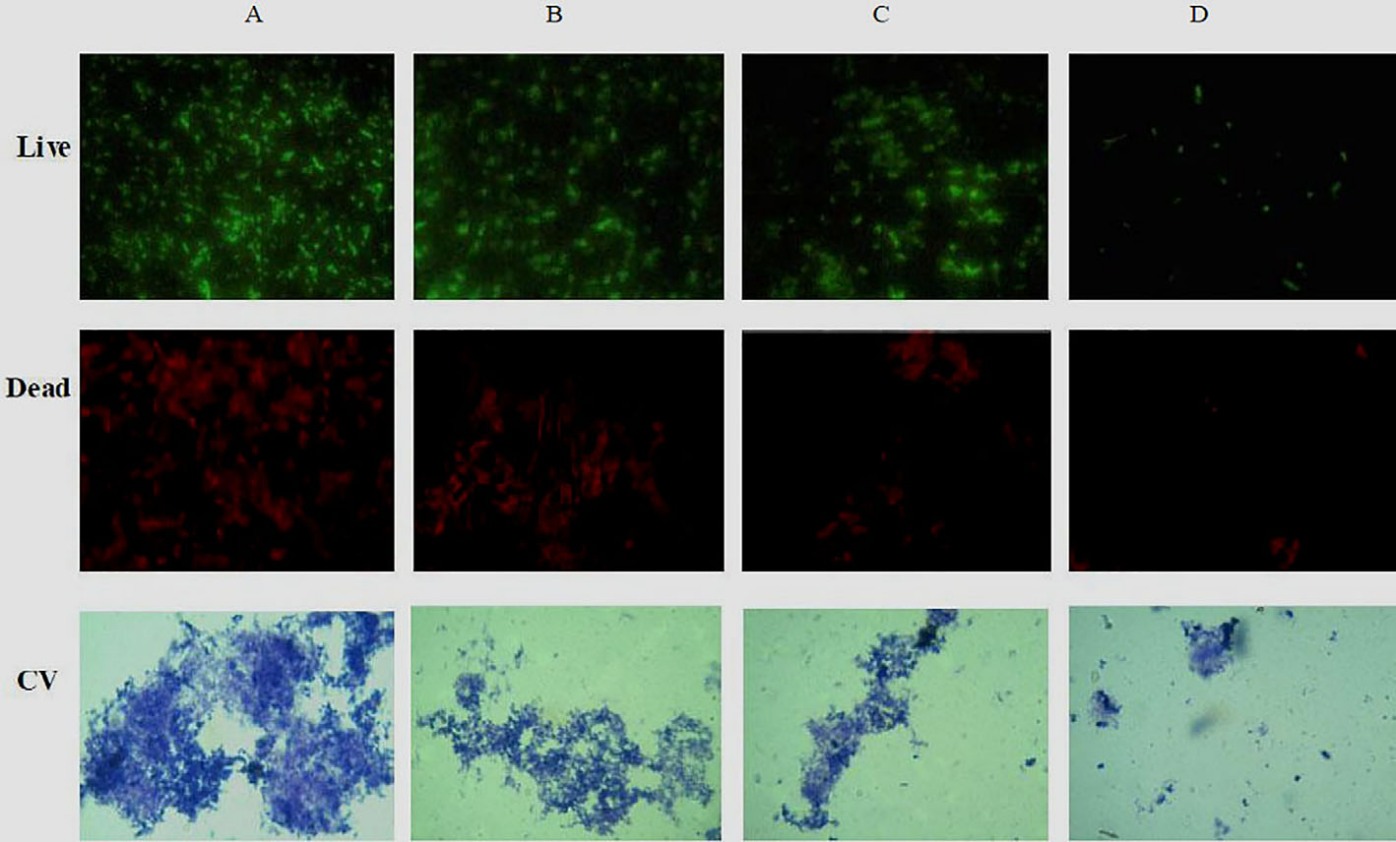
Fluorescence and light microscopic images of biofilms when treated with various concentrations of phlorotannins. **(A)** control, **(B–D)** demonstrated 1/8, 1/4 and 1/2 MIC phlorotannins treated, respectively.

The ability of phlorotannins to inhibit *P. aeruginosa* PAO1 biofilm formation activity was assessed using a crystal violet assay. As shown in **Figure 6**, a decrease in biofilm formation was observed when *P. aeruginosa* PAO1 was cultured with phlorotannins. Still, there were no statistically significant differences observed in reducing the biofilm formation at 0.0061–0.0121 g/ml. Similar results were also received by observing the biofilm with the help of light as well as fluorescent microscopy in **Figure 7**. It can be clearly observed that with the increase of phlorotannin concentration, the thinner biofilm appeared, which indicates intensive *P. aeruginosa* biofilm formation on coverslip surfaces. Viable bacteria cells were stained fluorescent green, whereas dead bacteria were stained fluorescent red. From the fluorescence micrograph images, we can find that with the increase of phlorotannin concentration, the green fluorescence and red fluorescence all decreased. Bacteria cell lysis at stationary phase, from **Figure 2**, we can find that at 10 h, *P. aeruginosa* cells were in the stationary growth phase; here we used 12 h culture to observe the biofilm, so the green fluorescence and red fluorescence all decreased at the same level. These results are in accordance with CV staining, which indicated the decreased biofilm biomass. Based on these results, it can be concluded that phlorotannins can inhibit biofilm formation.

### Phlorotannins Prevents *C. elegans* From Infection by *P. aeruginosa* PAO1

**Figure 8:**
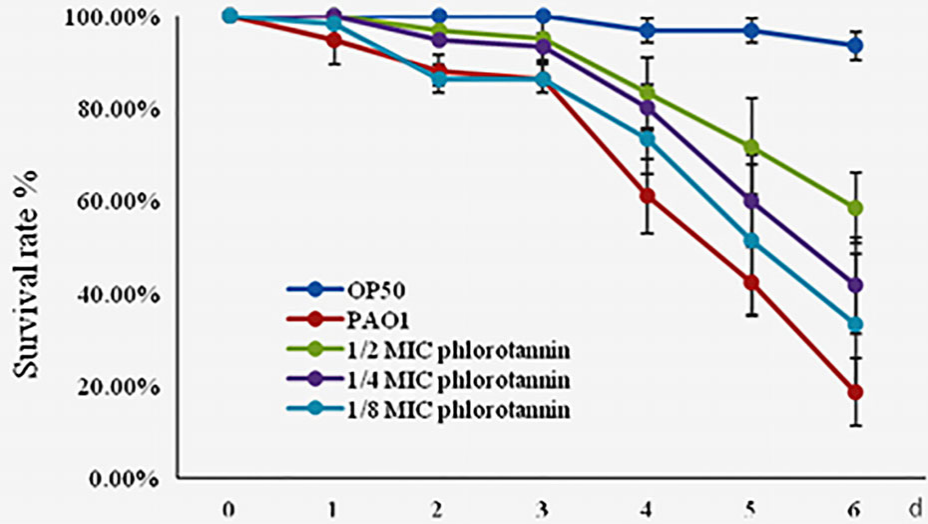
Effect of phlorotannins on the survival rate of *C. elegans* infected by *P. aeruginosa* PAO1. Results represent three independent experiments performed in triplicates.

Phlorotannins showed antibacterial and anti-QS activity *in vitro*; thus we detected their protective effect on *C. elegan*s against *P. aeruginosa* PAO1 infection. Phlorotannins increased the survival of the infected *C. elegans* (**Figure 8**); furthermore, phlorotannins reduced nematode death in a concentration-dependent manner.

## Discussion

Virulence factors are required as a critical condition for bacterial infection. Therefore, anti-virulence strategies have been developed to prevent bacterial infection instead of killing it using antibiotics. Interruption of the QS is a potential strategy to attenuate pathogenicity because virulence factor expressions are controlled by QS in many bacteria. Phlorotannins are marine polyphenols derived from brown algae. Phlorotannins possess broad range of biological properties such as antimicrobial, antiviral, anticancer, antioxidant, anti-inflammatory, anti-allergic, and anti-hyperpigmentation properties (Audibert et al., 2010; Lemesheva and Tarakhovskaya, 2018; Javed et al., 2020). In this study, phlorotannins were evaluated for their antibacterial and anti-QS activities. We found phlorotannins have antibacterial activity against selected gram-positive and negative bacteria *in vitro*, but phlorotannins have no synergistic effect with selected antibiotics. Pérez et al. (2016) reviewed the antimicrobial action of compounds from marine seaweed and their application in biofouling, aquaculture, and human health. Furthermore, Moubayed et al. (2017) found *Sargassum latifolium* B, *Sargassum platycarpum* A (collected from the red sea), and *Cladophora socialis* (collected from the Arabian Gulf) demonstrated antibacterial activity. The methanolic extracts of *Enteromorpha antenna*, *Enteromorpha linza*, and *Gracilaria corticata* possessed high total phenolic content and showed antimicrobial activity against food-borne pathogens except *P. aeruginosa* (Narasimhan et al., 2013).

Plant phenolic compounds have been reported have anti-QS activities. Violacein production by *C. violaceum* 12472 is regulated by the QS system. Therefore, the reduced production of violacein in *C. violaceum* is evidence of QS disruption (Javed et al., 2020). In this study, we found phlorotannins can reduce the purple pigment production in *C. violaceum* 12472. Our experimental data also showed that phlorotannins could decrease the virulence factor’s production and biofilm formation and also inhibit quorum sensing molecules production in *P. aeruginosa*. Moreover, phlorotannins also significantly improved the survival of *C. elegans* against *P. aeruginosa* infection. Our results agree with previous reports. Tea polyphenol inhibited biofilm formation and swimming motility of *Shewanella baltica* and also decreased extracellular protelytic activity, exopolysaccharide production in *S. baltica* (Zhu et al., 2015). Biancalani et al.’s study showed that polyphenolic extracts from vegetable residues had a high inhibitory activity on QS of *Pseudomonas savastanoi* (Biancalani et al., 2016). Red seaweed (*Chondrus crispus* and *Sarcodiotheca gaudichaudii*) extracts can suppress the expression of QS gene *sdiA*, reduce biofilm formation and motility, and down regulate the expression of genes encoding the virulence factors of *Salmonella enterica*. Red seaweed extract significantly increased the survival of the infected *C. elegans* (Kulshreshtha et al., 2016). Another study by Carvalho et al. (2017) showed that *Canistrocarpus cervicornis* extracts could inhibit QS of *C. violaceum* CV017 and inhibit bacterial attachment of *P. aeruginosa* PAO1. Jha et al. (2013) found that the extract from marine macro alga (*Asparagopsis taxiformis*) has QS inhibition properties, and the expected active compound was 2-dodecanoyloxyethanesulfonate (C_14_H_27_O_5_S). Batista et al. (2014) showed that 20 polar extracts of macroalgae from Arraial do Cabo, Brazil, interrupted the QS of the reporter *C. violaceum*. Halogenated furanones from the marine algae *Delisea pulchra* inhibit AHLs-mediated quorum sensing (Gram et al., 1996). Three AHL inhibitors have been isolated from the Korean red alga *Ahnfeltiopsis flabelliformis* (Kim et al., 2007).

Based on our results, it is proven that phlorotannins possess anti-QS activities and can attenuate virulence determinants of *P. aeruginosa*. However, future investigations should use the phlorotannins in animal feed to evaluate its clinical effect.

In conclusion, phlorotannins can inhibit the QS activity, reduce virulence factor’s production, and disrupt biofilm formation, which is crucial to the pathogenicity in *P. aeruginosa*. Phlorotannins present its use as an important natural source for controlling bacterial infections in the future. This method provides a viable alternative strategy to antibiotics against pathogens, without selective pressure on the drug resistant mutant strains and can be used in pharmaceutical industries.

## Data Availability Statement

The original contributions presented in the study are included in the article/supplementary material. Further inquiries can be directed to the corresponding author.

## Author Contributions

WC designed the research. JT and WW performed sampling and performed experimental work. WW performed data analysis. WC wrote the manuscript and input from all authors. All authors contributed to the article and approved the submitted version.

## Funding

This research was funded by the Priority Academic Program Development of Jiangsu Higher Education Institutions (PAPD).

## Conflict of Interest

The authors declare that the research was conducted in the absence of any commercial or financial relationships that could be construed as a potential conflict of interest.
